# Influence of *Moraxella *sp. colonization on the kidney proteome of farmed gilthead sea breams (*Sparus aurata*, L.)

**DOI:** 10.1186/1477-5956-8-50

**Published:** 2010-10-12

**Authors:** Maria Filippa Addis, Roberto Cappuccinelli, Vittorio Tedde, Daniela Pagnozzi, Iolanda Viale, Mauro Meloni, Fulvio Salati, Tonina Roggio, Sergio Uzzau

**Affiliations:** 1 Porto Conte Ricerche Srl, Tramariglio, Alghero (SS), Italy; 2 Dipartimento di Patologia e Clinica Veterinaria, Università degli Studi di Sassari, Sassari, Italy; 3 State Veterinary Institute, IZS of Sardinia, Oristano, Italy; 4 Dipartimento di Scienze Biomediche, Università degli Studi di Sassari, Sassari, Italy

## Abstract

**Background:**

Currently, presence of *Moraxella *sp. in internal organs of fish is not considered detrimental for fish farming. However, bacterial colonization of internal organs can affect fish wellness and decrease growth rate, stress resistance, and immune response. Recently, there have been reports by farmers concerning slow growth, poor feed conversion, and low average weight increase of fish farmed in offshore floating sea cages, often associated with internal organ colonization by *Moraxella *sp. Therefore, presence of these opportunistic bacteria deserves further investigations for elucidating incidence and impact on fish metabolism.

**Results:**

A total of 960 gilthead sea breams (*Sparus aurata*, L.), collected along 17 months from four offshore sea cage plants and two natural lagoons in Sardinia, were subjected to routine microbiological examination of internal organs throughout the production cycle. Thirteen subjects (1.35%) were found positive for *Moraxella *sp. in the kidney (7), brain (3), eye (1), spleen (1), and perivisceral fat (1). In order to investigate the influence of *Moraxella *sp. colonization, positive and negative kidney samples were subjected to a differential proteomics study by means of 2-D PAGE and mass spectrometry. Interestingly, *Moraxella *sp. infected kidneys displayed a concerted upregulation of several mitochondrial enzymes compared to negative tissues, reinforcing previous observations following lipopolysaccharide (LPS) challenge in fish.

**Conclusions:**

Presence of *Moraxella *sp. in farmed sea bream kidney is able to induce proteome alterations similar to those described following LPS challenge in other fish species. This study revealed that *Moraxella *sp. might be causing metabolic alterations in fish, and provided indications on proteins that could be investigated as markers of infection by Gram-negative bacteria within farming plants.

## Background

The occurrence of a bacterial infection outbreak in an aquaculture plant can produce important detrimental effects, including decreased growth rate, impaired fish wellness, and poor productivity, and may rapidly lead to widespread fish losses if left uncontrolled [1]. Therefore, the tanks or cages need to be constantly monitored for absence of potentially deleterious bacteria. However, several bacterial species isolated from fish tissues, including *Moraxella *[2], do not currently receive attention as potentially harmful for the fish farming economy, although infections by *Moraxella *sp. are becoming increasingly common in Mediterranean aquaculture plants. There have been increasing reports from farmers concerning slow fish growth, losses in production efficiency, and poor feed conversion, which fail to be associated to presence of known bacterial pathogens. Such problems might be associated to colonization by opportunistic bacterial species that, although not causing clinically evident pathogenic effects, do impair production efficiency. In many of these occurrences, *Moraxella *sp. was isolated from internal organs of fish within the problematic farm (Fulvio Salati, unpublished observations). In our opinion, this finding deserves further attention, and investigations are needed in order to assess its biological significance and evaluate its consequences on aquaculture production. In fact, although detection of *Moraxella *sp. in fish has not yet been associated to infection outbreaks with significant economical losses for aquaculture plants, presence of bacteria in internal organs of fish may reduce fish wellness and growth, and result in symptomatic infections under stressful conditions [3]. Moreover, Corbeil *et al. *[4] demonstrated that presence of *Moraxella *sp. can also enhance the growth of other bacterial pathogens, such as *Mannheimia haemolytica*, *Pasteurella multocida*, and *Histophilus somni*.

*Moraxella *sp. can be directly responsible for symptomatic disease in different host species. In cattle, for instance, *M. bovis *is the most commonly recognized cause of infectious keratoconjunctivitis (pink eye) [5], whereas other species such as *M. bovoculi *and *M. ovis *are increasingly associated to conjunctivitis in cattle and small ruminants [6-8]. In humans, although long considered as a harmless commensal, *Moraxella *sp. is now commonly accepted as a pathogen responsible for otitis media, sinusitis and, occasionally, laryngitis [9]. In particular, *M. catarrhalis *is associated with nosocomial infections. It can act as an "opportunistic pathogen" in the immunocompromised host and in patients with underlying chronic lung disease, causing bronchitis, pneumonia and, occasionally, bacteremia and meningitis [10-14]. Apart from the consequences deriving from specific pathogenetic mechanisms, non-specific responses can also be produced on the host by the lipopolysaccharide (LPS) of Gram-negative bacteria, that acts as a non-specific endotoxin; different authors explored the consequences of LPS administration in fish, with interesting results [15,16].

In order to elucidate the influence of internal organ colonization by *Moraxella *sp. in economically relevant fish, internal organs of farmed and wild gilthead sea bream (*Sparus aurata*, L.) were monitored for presence of pathogens throughout the production cycle. Individuals positive for *Moraxella *sp. were subjected to a proteomic study in order to investigate the consequences of bacterial colonization on the host tissue metabolism.

## Results

### Presence of *Moraxella *sp. in gilthead sea bream

A large survey was performed in order to evaluate the health status of sea breams produced in four farming plants based on offshore floating sea cages located around the coastal area of Sardinia, plus two natural populations from lagoons. Within this study, internal organs of 960 seabreams were subjected to routine microbiological examination. Positivity to bacteria identified as *Moraxella *sp. was detected in fish from different locations (Table 1). The prevalence (P) of *Moraxella *sp., calculated as the number of individuals infected/number of hosts examined, was of 1.35% (13 fish out of 960). Presence of *Moraxella *sp. did not show evident correlations to temperature, salinity, season, or size of fish, and it was recorded throughout the year both in offshore cages and in natural lagoons.

**Table 1:** Summary of data on individuals positive to *Moraxella *sp.

	Date	Water parameters			Case No. and Localization	Parameters (mean cage parameters for 100 fish)		
				
		Sal (‰)	T (°C)	**O**_**2 **_**(mg/L)**		L (cm)	W (g)	K
A	03Oct07	37	21.5	6.2	1. Eye	12.2 (12.0 ± 1.1)	28.1 (27.1 ± 6.8)	1.55 (1.52 ± 0.08)

B	17Nov07	37	18.5	6.7	1. Perivisceral fat	24.0 (19.2 ± 1.3)	216.0 (126.0 ± 30.4)	2.03 (1.75 ± 0.12)

A	19Dec07	36	12.4	6.8	1. Kidney	13.3 (13.4 ± 0.8)	35.7 (35.6 ± 6.7)	1.52 (1.47 ± 0.08)

A	05Mar08	37	13.0	6.8	1. Kidney	14.4 (14.3 ± 1.0)	43.5 (44.0 ± 9.3)	1.43 (1.48 ± 0.09)

C	17Mar08	37	14.5	6.8	1. Kidney	27.2 (26.0 ± 1.7)	327.0 (272.3 ± 60.3)	1.63 (1.53 ± 0.10)

					2. Spleen	25.3 (26.0 ± 1.7)	230.0 (272.3 ± 60.3)	1.42 (1.53 ± 0.10)

B	01Apr08	36	14.5	6.7	1. Kidney	19.5 (20.6 ± 1.2)	125.6 (157.7 ± 31.9)	1.69 (1.79 ± 0.10)

					2. Brain	19.7 (20.6 ± 1.2)	147.8 (157.7 ± 31.9)	1.93 (1.79 ± 0.10)

					3. Brain	20.6 (20.6 ± 1.2)	161.1 (157.7 ± 31.9)	1.84 (1.79 ± 0.10)

D	29Aug07	37	26.8	6.2	1. Kidney	25.3 (26.2 ± 1.7)	249.0 (290.7 ± 60.1)	1.54 (1.59 ± 0.08)

E	23Oct07	30	24.7	5.8	1. Brain	24.4 (26.6 ± 1.2)	225.4 (276.6 ± 42.5)	1.55 (1.49 ± 0.08)

					2. Kidney	28.4 (26.6 ± 1.2)	353.9 (276.6 ± 42.5)	1.54 (1.49 ± 0.08)

					3. Kidney	26.7 (26.6 ± 1.2)	299.1 (276.6 ± 42.5)	1.57 (1.49 ± 0.08)

Maricultured; A, B, C, D. Wild; E. Sal: salinity; T: temperature; O_2_: dissolved Oxygen; L: length; W: weight; K: condition factor = (weight × 100)/total length^3^.

Positivities are grouped according to date of sampling and location of the farming plant. Environmental and zootechnical parameters are reported. Data in parentheses indicate the mean parameters of all fish within the floating cage where sampling was performed.

Bacterial colonization was detected mainly in kidney (7), followed by brain (3), eye (1), spleen (1), and perivisceral fat (1). As far as samplings are concerned, kidney positivity was present in 6 out of 8 positive samplings. Moreover, when more than one individual in a cage or lagoon was colonized by *Moraxella *sp., kidney was always involved (Table 1). Twelve positive fish out of 13 were apparently healthy, and size, weight, and K index were within 1SD from the mean. The seabream with eye positivity, on the contrary, suffered a serious exophtalmus. This was particularly interesting since colonization by *Moraxella *sp. is often associated to conjunctivitis in different mammalian host species.

### 2-D PAGE and image analysis of kidney tissue

Being the organ most affected by *Moraxella *sp. colonization in this study, kidney was investigated for presence of biochemical markers related to presence of these bacteria. A gel-based proteomic approach was chosen for this study, since it provides a top-down, comprehensive picture of the total protein expression pattern of a tissue, and enables its comparison among different physiological and pathological states. This is especially valuable when scarce information is available about an organism or tissue proteome, as is the case of gilthead sea bream and most fin fish. In fact, in the scientific literature there are virtually no proteome expression data on sea bream kidney, and proteomic data on fish kidney in general are very scarce.

**Figure 1:**
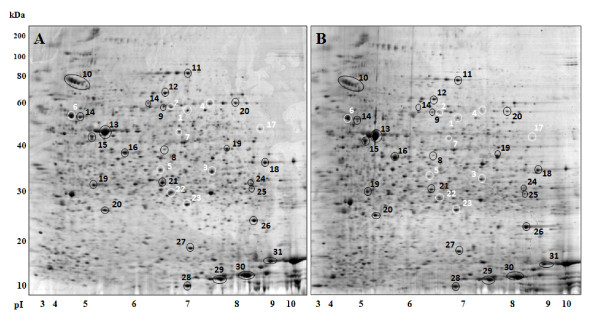
**2-D PAGE map of *Moraxella *spp. positive (A) and negative (B) kidney tissue**. Circled, numbered spots indicate all spots identified in this work, ordered following the increase in expression upon *Moraxella *sp. colonization. The ten spots most significantly upregulated in positive kidney are indicated in white. Protein identifications corresponding to spot numbers are reported in Table 2.

All 7 positive kidney tissue samples plus 28 negative kidney tissue samples (n = 35; 5 fish from each of the 6 samplings showing at least one positivity to *Moraxella *sp.) were subjected to 2-D PAGE analysis. After optimization of the extraction and focusing protocol, a good coverage of the kidney proteome expression pattern was obtained. Representative 2-D PAGE maps of *Moraxella *sp. positive (A) and negative (B) kidney tissue are reported in Fig. 1.

The 10 best resolved 2-D maps of kidney tissue were selected for image analysis (3 maps from positive samples, 1 from farm D and 2 from the natural population lagoon E, and 7 maps from negative samples, 4 from farm D and 3 from the natural population lagoon E). A very high number of spots (about 600 per map) was generally observed in all maps selected for image analysis, especially when considering that a medium-sensitivity, mass-spectrometry compatible stain was used. The protein maps appeared well balanced in terms of relative expression. A predominant expression level could be observed only for a few high-abundance proteins, which however did not hamper generation of well-focused and resolved maps. Upon image analysis with ImageMaster 2D Platinum V. 6.0, protein patterns were highly reproducible, and spots could be easily matched among all selected maps. The matching statistics for 2-D maps from different individuals were around 60%. After definition of landmarks, elimination of erroneously detected spots, deletion of wrong matches, and correction of missed matches, the match statistics report rose to 75%.

### Differential expression of kidney proteins upon colonization by *Moraxella *sp

The differential analysis of protein expression among tissue maps of *Moraxella*-positive and negative kidneys revealed the differential expression of several protein spots. In particular, ten spots were prominently upregulated in *Moraxella*-positive tissue and reproducibly underexpressed in negative tissues (Fig. 1, in white). These spots were subjected to MALDI-MS and nanoHPLC-nanoESI-Q-TOF-MS/MS for identification, producing the following matches: mitochondrial alanine aminotransferase, mitochondrial aldehyde dehydrogenase, mitochondrial dihydrodipicolinate synthase, mitochondrial methylmalonate semialdehyde dehydrogenase, mitochondrial ATP synthase beta subunit, mitochondrial Acyl-CoA-dehydrogenase, S-adenosyl-homocysteine hydrolase, and peroxiredoxins. Interestingly, seven out of ten spots with a statistically significant upregulation in *Moraxella*-positive kidney produced identifications corresponding to mitochondrial enzymes (Table 2).

**Table 2:** Proteins identified in sea bream kidney tissue.

**N**.	Protein	**Acc. no**.	Species	Pred. mass	Pred. pI	**Sc**.	QM (%c.)	FC	*p*
1	**Alanine aminotransferase, mt**	gi|37783307	*S. aurata*	62091	8.54	527	26(23)	**2.3**	**0.0016**
2	**Aldehyde dehydrogenase, mt**	gi|118503	*E. caballus*	54532	5.7	76	2(4)	**3.3**	**0.0005**
3	**Dihydrodipicolinate synthase, mt**	gi|47208001	*T. nigroviridis*	34365	8.17	100	9(5)	**1.6**	**0.0001**
4	**Methylmalonate semialdehyde dehydrogenase, mt**	gi|47230188	*T. nigroviridis*	57397	7.06	410	10(12)	**2.3**	**0.0005**
5	**Dihydrodipicolinate synthase, mt**	gi|47208001	*T. nigroviridis*	34365	8.17	110	9(6)	**2.2**	**0.0006**
6	**ATP synthase, beta subunit, mt**	gi|47605558	*C. carpio*	55327	5.05	1432	76(46)	**1.5**	**0.005**
7	**S-adenosyl-homocysteine hydrolase**	gi|178277	*H. sapiens*	48254	6.03	156	7(6)	**2.6**	**0.0104**
8	Phosphoenolpyruvate carboxykin.	gi|24637098	*S. aurata*	14574	5.61	48	1(9)	<1.5	>0.05
9	*Antiquitin*	gi|61742178	*A. schlegelii*	55832	5.88	369	9(13)	*1.71*	*>0.05*
10	*Wap65*	gi|119393859	*A. schlegelii*	49162	5.40	250	11(10)	*1.86*	*>0.05*
11	*Transferrin*	gi|34329603	*A. schlegelii*	76152	6.38	418	12(9)	*2.69*	*>0.05*
12	Transferrin	gi|33113484	*P. major*	76146	5.72	206	6(6)	<1.5	>0.05
13	Beta actin	gi|33526989	*M. albus*	42110	5.31	752	41(45)	<1.5	>0.05
14	ATP synthase, mt	gi|66773080	*D. rerio*	55080	5.25	1546	79(47)	<1.5	>0.05
15	Cytoplasmic actin	gi|13699190	*L. japonicum*	42137	5.30	777	57(42)	<1.5	>0.05
16	Beta actin	gi|49868	*M. musculus*	39446	5.78	557	59(28)	<1.5	>0.05
17	**Acyl-Co A dehydrogenase, mt**	gi|47209002	*T. negroviridis*	39802	6.08	211	4 (11)	**1.9**	**0.0117**
18	Fructose-biphosphate aldolase B	gi|1703243	*S. aurata*	40190	8.43	664	25(20)	<1.5	>0.05
19	Electron transfer flavopr. alpha, mt	gi|47225813	*T. nigroviridis*	35017	7.64	406	14(26)	<1.5	>0.05
20	PEBP superfamily	gi|47221502	*T. nigroviridis*	21069	6.89	393	20(27)	<1.5	>0.05
21	*Carbonic anhydrase*	gi|56554783	*P. americanus*	28512	5.22	64	4(10)	*2.21*	*>0.05*
22	**Peroxiredoxin**	gi|47220267	*T. nigroviridis*	22280	5.44	313	24(22)	**2.5**	**0.0025**
23	**Peroxiredoxin family protein**	gi|93211500	*P. maxima*	22063	5.58	364	21(32)	**1.6**	**0.05**
24	Enoyl-CoA hydratase short chain	gi|12805413	*M. musculus*	31636	8.51	224	13(12)	<1.5	>0.05
25	*Glutathione S-transferase*	gi|34014736	*S. aurata*	24748	8.51	205	10(28)	*1.6*	*>0.05*
26	Nucleoside diphosphate kinase	gi|10121713	*G. mirabilis*	17214	8.52	299	39(48)	<1.5	>0.05
27	*Cu/Zn superoxide dismutase*	gi|62550923	*S. aurata*	6979	5.41	545	25(68)	*1.8*	*>0.05*
28	Alpha-2 globin	gi|99122203	*S. aurata*	15887	8.79	96	11(6)	<1.5	>0.05
29	Beta globin	gi|91260232	*S. aurata*	16308	7.82	158	7(18)	<1.5	>0.05
30	Alpha-2 globin	gi|99122203	*S. aurata*	15887	8.79	466	23(41)	<1.5	>0.05
31	Beta globin	gi|91260232	*S. aurata*	16308	7.82	367	10(47)	<1.5	>0.05

N, spot number; Acc. no., accession number; Pred. mass, predicted mass; Pred. pI, predicted pI; Sc, score; QM (%c.), Queries matched (% coverage); FC, fold change.

Protein identifications were performed both by MALDI-MS and nano-HPLC-nano-ESI-Q-TOF-MS/MS. Proteins with fold change > 1.5 and P < 0.05 are in bold. Proteins with fold change > 1.5 and *p *> 0.05 are in italics.

Several other protein spots showed an increase of expression levels higher than 1.5 fold in Moraxella-positive kidney, but *p *values were higher than 0.05. Although less relevant as indicators of an infection state, these proteins were subjected to mass spectrometry identification in order to gain further knowledge on the influence of bacterial colonization in fish kidney metabolism, together with other few proteins with fold changes slightly lower than 1.5 (Fig. 1). The highest abundance spots, expectedly related to actin and globins (n = 6), were also excised and subjected to identification as controls. After MS analysis, 31 proteins were identified, and are listed in Table 2 according to the numbering indicated in Fig. 1. Table 2 reports also fold changes and *p *values for all protein spots considered in this study.

## Discussion

Several indicators suggest that sea bream colonization by bacteria belonging to *Moraxella *species might be increasing in Mediterranean mariculture plants, and that presence of these bacteria in internal organs of farmed fish might be linked to slow growth, reduced wellness, and higher susceptibility to other diseases. In this study, the prevalence of internal organ positivity to *Moraxella *sp. was found to be of 1.35% on almost 1,000 fish examined. *Moraxella *sp. colonization was detected throughout the year, in all locations examined, and at different temperatures, both in floating offshore cages and lagoons (Table 1). In general, fish colonization was not associated to evident symptoms, with the exception of a severe exophtalmus observed upon eye positivity. Eye symptoms are consistent with infection by *Moraxella *sp. in other animal species and in humans. Therefore, *Moraxella *sp. might be causing ocular damages (i.e. exerting direct pathogenic effects) also in fish, although presence of other underlying conditions could not be ruled out completely.

Many diseases of fish have a multifactorial origin, and might stem from presence of an aetiological agent combined with environmental stresses. As such, *Moraxella *sp. might be considered an opportunistic bacterial pathogen, which could cause illness in "stressed" fish. Indeed, colonization of internal organs might impair the physiological tissue functions and render fish more vulnerable to the environment or to the occurrence of infections by other pathogens [3,4]. Moreover, as reported in other organisms, *Moraxella *sp. might act as an opportunistic pathogen in fish under acute or chronic stressful conditions, with increased susceptibility to other infectious diseases. In both circumstances, the identification of biochemical markers enabling identification of an early host response to asymptomatic infections by *Moraxella *sp. and/or other opportunistic Gram-negative pathogens appears of considerable interest. The availability of a preclinical marker would be welcome for detecting colonization and for containing bacterial infection outbreaks before they spread to the whole cage or plant, especially when uncontrolled, wild fish are used for restocking.

The molecular approach used in this study offered us the opportunity to investigate the protein expression profile of healthy, bacteria-free kidneys, and to evaluate the changes that renal tissue undergoes when Gram-negative bacteria are present. Higher relevance was given to proteins with a marked upregulation in the infected kidney, in order to pinpoint proteins with potential as markers of Gram-negative infections, which could be used as tools to quickly detect the menace of an outbreak in the farming plant and enable intervention within short time frames, avoiding loss of fish.

In *Moraxella*-positive sea bream kidney tissue, seven out of ten proteins showing a statistically significant upregulation were mitochondrial enzymes (Table 2, bold). This was an interesting finding, since mitochondrial proteins are central to various metabolic activities and are key regulators of apoptosis. Notably, disturbance of mitochondrial proteins is often associated with disease [17].

Interestingly, other non-mitochondrial proteins significantly upregulated in *Moraxella-*positive kidney tissue were peroxiredoxins and S-adenosyl-homocysteine hydrolase, together with indications about upregulation of antiquitin, Warm acclimation-related protein 65, transferrin, glutathione S-transferase, carbonic anhydrase, and Cu/Zn superoxide dismutase, many of which are known to be related to cellular responses to oxidative stress, infection, inflammation, or programmed cell death processes. Transferrin was also found to be significantly upregulated in rainbow trout serum following intraperitoneal inflammation and LPS injection [16].

The number of fish used to gather proteomic data in this study was limited, and variations in expression levels of mitochondrial proteins might as well have been induced by other metabolic perturbations, or be dependent on underlying alterations due to non-infectious factors. This notwithstanding, the value of the enzymatic markers proposed in this study is enhanced and reinforced by the fact that our findings, observed in naturally occurring infections by *Moraxella *sp., are consistent with the data reported by Roher and coworkers [15] following experimental administration of LPS. These authors investigated the physiological consequences of administrating LPS to rainbow trout. Important changes in metabolic, mitochondrial, and structural genes were observed according to tissue metabolism. In aerobic tissues, that obtain energy mainly from oxidative phosphorylation, LPS provoked marked changes in representative mitochondrial genes, whereas in anaerobic tissues major expression changes were observed in glycolytic enzymes. The findings reported by these authors reinforce the considerations about the potential detrimental effects of internal organ colonization by Gram-negative bacteria, including opportunistic pathogens, on growth and metabolic processes of fish.

## Conclusions

This study reports, for the first time and through a differential proteomic investigation of the seabream kidney tissue, that naturally occurring fish infections by Gram-negative bacteria considered as being opportunistic pathogens can alter the expression of mitochondrial enzymes in infected kidney tissues, and therefore produce a condition of stress that has the potential to alter farmed fish metabolism. A panel of up-regulated enzymes was identified in tissues infected by *Moraxella *sp. The change in expression levels of these proteins could be investigated as a marker of infection by frankly pathogenic Gram-negative bacteria, as well as for the identification of an early host response to asymptomatic infections by opportunistic Gram-negative pathogens in fin fish under stressful conditions.

## Methods

### Gilthead sea bream farming plants and sampling conditions

Gilthead sea bream samplings were performed in 4 offshore sea cage plants and in two natural populations from lagoons. Every two months, for 17 months in total (the entire production cycle, from seeding to commercial size), fish were sampled randomly from each plant, immersed in marine water and ice, and transported to the laboratory. For every sampling, water temperature, salinity, and dissolved oxygen were measured with an immersion thermometer, an optical refractometer, and a HQ 20 Hach Portable LDO™ Dissolved Oxygen Meter (Hach Company, Leveland, CO, USA), respectively. Once in the laboratory, fish were measured, weighed, and the condition factor K = (weight × 100)/total length^3 ^was calculated. Internal organs were collected and stored at -80°C until needed.

### Ichthyopathological examination

A total of 960 fish were examined, 840 from floating sea cages and 120 from natural lagoons. Gross-anatomy, parasitological and microbiological examinations were carried out to reveal the presence of diseases. Microbiological examination was carried out by classical methods as previously described [18]. In particular, bacterial isolation was carried out by loop on Brain Heart Infusion (BHI) (Difco Laboratories, Detroit, MI, USA) Agar plates added with 1.0% NaCl and incubated at 20°C. Isolates from kidney, brain, eyes, and perivisceral fat on BHI agar were examined by optical microscopy after Gram staining, and subjected to routine tests for the determination of biochemical characteristics as described in the Manual of Methods for General Bacteriology [19]. Classification in groups and bacterial strain identification was made on the basis of morphological and biochemical characteristics of the isolates and by means of API Galleries 20E or 20NE (bioMérieux, Roma, Italy). Then, identification of *Moraxella *sp. was confirmed by PCR, using the primers designed on the ITS of *M. catarrhalis *strain BCRC 10630 16S-23S ribosomal RNA, complete sequence EU014605.1. In particular, PCR was carried out in a reaction mixture containing 3.0 mM MgCl_2 _(Invitrogen, San Diego, CA), 0.2 μM of each primer, 0.5 mM of dNTPs (dCTP, dGTP, dATP, dTTP) (Invitrogen), 1 U of Platinum *Taq *DNA Polymerase (Invitrogen), 1 μl of the extracted DNA and nuclease free water up to 25 μl of final volume. MM19 (5'-ATA ACG CGG GGG TCA TAA GTT-3') and MM20 (5'-GTT TAC GCT TGA TTC AGT TCT CTT-3') were used as forward and reverse primer respectively in order to amplify an approximate 330 bp target region within the 16S -23S rRNA intergenic spacer. They were synthesized by MWG Biotech (Germany). PCR was performed in an MJ Mini thermal cycler (Biorad Laboratories, Berkeley, CA) using the following thermal parameters: 2 min at 95°C, then 40 cycles of 30 s at 95°C, 30 s at 51°C and 1 min at 72°C, followed by a final elongation of 7 min at 72°C. A 2% SYBR Safe (Invitrogen) stained agarose gel was used to evaluate the correct amplification of the target; 7 μl of the amplification product were visualised with the Safe Imager and the PhotoDoc-It Imaging System (Invitrogen).

### Preparation of protein extracts

Protein extracts were obtained from frozen kidney tissues as follows. Half organ was minced with a sterile scalpel and 100 mg of tissue were placed in 2 mL Eppendorf safe-lock tubes (Eppendorf, Hamburg, Germany), immersed at 5% w/v in lysis buffer (8M urea, 4% CHAPS, 0.5% IPG buffer, GE Healthcare, Little Chalfont, UK), and subjected to three cycles of 1.5 min at 30 cycles/s in a TissueLyser mechanical homogenizer (Qiagen, Hilden, Germany). All extracts were clarified for 15 min at 12,000 × g at 4°C, quantified by the Bradford method [20], checked for quality and quantity by SDS-PAGE, and then stored at -80°C until needed.

### Protein electrophoresis and image analysis

SDS-PAGE was performed according to Laemmli [21] on 10% polyacrylamide gels on a Protean Tetra Cell (Bio-Rad) following the manufacturer's instructions, and gels were stained with the colloidal Coomassie stain [22]. For 2-D PAGE, proteins were diluted in DeStreak rehydration solution (GE Healthcare). Resuspended proteins (600 μg) were absorbed overnight into 24 cm IPG strips (GE Healthcare, pH 3-10 NL), and focused on an IPGPhor instrument (GE Healthcare) for a total of 80,000 Vh. After focusing, strips were equilibrated in 50 mM Tris HCl, pH 6.8, 2% SDS, 7 M urea, 10% glycerol, supplemented with 2% DTT for 15 min, and then with 2.5% iodoacetamide for 15 min. The second dimension (SDS-PAGE) was performed on 8% to 16% polyacrylamide gradient gels, on an Ettan DALTsix electrophoresis system (GE Healthcare), following the manufacturer's instructions. 2-D gels were stained with the colloidal Coomassie method [22] and images were digitalized with an Image Scanner (GE Healthcare). Digitalized images were then processed with ImageMaster Platinum 6.0 (GE Healthcare) for comparison of protein spots. Images were subjected to an image analysis workflow consisting of spot detection, spot matching, and differential expression analysis. All steps performed by the software were manually checked in order to eliminate errors such as artifactual spots, merged spots, and missed spots. For comparative analysis, %Vol values of spots were compared among the different 2D maps using the default parameters of ImageMaster Platinum 6.0. The data generated by ImageMaster for upregulated spots were used for calculating mean and SD of the %Vol for each spot within the two groups (positive and negative samples), and plotted with Microsoft Excel (Microsoft Corp., Redmond, WA). Statistical significance of differences among the two groups (positive and negative samples) was also assessed by the two-tailed independent groups *t*-test for means with a 95% confidence level, and *p *values were calculated.

### Spot picking and in-situ tryptic digestion

Protein spots were manually excised from gels, destained with 15 mM K_3_Fe(CN)_6 _in 50 mM Na_2_S_2_O_3_, washed with water, and stored in acetonitrile. Spots were then subjected to an O/N tryptic digestion at 37°C in 50 mM NH_4_HCO_3_, pH 8.0, using 40 to 100 ng of trypsin depending on spot intensity. Peptide mixtures were collected by squeezing with acetonitrile followed by centrifugation. Peptides were then acidified with TFA 20%, dried in SpeedVac^®^, resuspended in 0.2% formic acid and stored at -20°C.

### Mass Spectrometry

#### MALDI-MS

MALDI-MS analysis was performed as described previously [23]. Briefly, peptide mixture was mixed with matrix solution on target plate, and allowed to dry at room temperature. MALDI-mass spectra were recorded with a MALDI micro MX mass spectrometer (Waters, Manchester, UK) equipped with a reflectron analyzer and used in delayed extraction mode. Mass calibration was performed by using the standard mixture provided by manufacturer. Raw data, reported as monoisotopic masses, were then introduced into in house MASCOT peptide mass fingerprinting search program (Version 2.2, Matrix Science, Boston, MA), and used for protein identification.

#### NanoHPLC-nanoESI-Q-TOF-MS

LC-MS/MS analyses were performed as described previously [24] on a Q-TOF hybrid mass spectrometer equipped with a nano lock Z-spray source, and coupled on-line with a capillary chromatography system CapLC (Waters). After loading, the peptide mixture was first concentrated and then washed onto a reverse-phase pre-column (Symmetry 300, C18, 5 μm, NanoEase, Waters). The sample was then fractionated onto a C18 reverse-phase capillary column (Nanoflow column 5 μm Biosphere C18, 75 μm × 200 mm, Nanoseparations). The mass spectrometer was set up in a data-dependent MS/MS mode where a full scan spectrum was followed by tandem mass spectra. Peptide ions were selected as the three most intense peaks of the previous scan. Suitable collision energy was applied depending on the mass and charge of the precursor ion. Argon was used as the collision gas. ProteinLynx software, provided by the manufacturers, was used to analyze raw MS and MS/MS spectra and to generate a peak list which was introduced in the in-house Mascot MS/MS ion search software for protein identification.

## Competing interests

The authors declare that they have no competing interests.

## Authors' contributions

MFA performed the experimental design, carried out 2D electrophoresis, performed image analysis and statistical analysis, and drafted the manuscript. RC performed all fish samplings, collected environmental data, and coordinated the interaction with fish farmers. VT performed sample preparation, assisted with 2D electrophoresis, performed excision and digestion of protein spots, and carried out MALDI-MS. DP carried out HPLC-nano-ESI-Q-TOF-MS/MS and provided guidance with MALDI-MS. IV performed microbiological examinations. MM performed molecular identifications. FS performed ichtyopathological examinations, coordinated the microbiology and molecular biology assays, and helped to draft the manuscript. TR participated in coordination of the study and data interpretation, and helped to draft the manuscript. SU conceived the study, participated in its design and coordination, and helped to draft the manuscript. All authors read and approved the final manuscript.
